# Influence of anonymous HIV testing on national HIV surveillance in the Republic of Korea (2000 to 2015): a retrospective analysis

**DOI:** 10.1186/s12889-019-7866-y

**Published:** 2019-11-27

**Authors:** Mee-Kyung Kee, Myeongsu Yoo, Jaehyung Seong, Ju-Yeon Choi, Myung Guk Han, Joo-Shil Lee, Youngmee Jee, Kisoon Kim, Sung Soon Kim, Chun Kang

**Affiliations:** 1 Division of Viral Disease Research, Center for Infectious Disease Research, Korea National Institute of Health, Cheongju-si, South Korea; 2 Division of Viral Diseases, Center for Laboratory Control of Infectious Diseases, Korea Centers for Diseases Control and Prevention, Cheongju-si, South Korea; 3 Government-wide R&D Fund for Infectious Disease Research, Cheongju-si, South Korea; 4 Center for Infectious Disease Research, Korea National Institute of Health, Cheongju-si, South Korea; 5 Division of Bacterial Disease Research, Center for Infectious Disease Research, Korea National Institute of Health, Cheongju-si, South Korea

**Keywords:** HIV prevalence, Anonymous test, Public health center, Korea

## Abstract

**Background:**

Owing to the continuous increase in the number of new human immunodeficiency virus (HIV) infection in Korea, public health centers (PHCs) have performed anonymous tests since 1989. No study has examined the patterns of anonymous HIV testing performed at PHCs and the characteristics of HIV infection detected in those tests. We aimed to assess the influence of anonymous HIV testing on Korea’s national HIV surveillance.

**Methods:**

HIV screening test data from 253 PHCs over a 16-year period were classified into 13 groups based on reason for testing. For anonymous HIV test takers (Anonymous), the HIV positivity per 10,000 tests was calculated, as repetitions could not be distinguished. Those with suspected HIV infection voluntarily underwent HIV testing and revealed their identity (Suspected). HIV prevalence was calculated as the number of HIV-positive persons per 10,000 test takers. Analyses were performed using chi-square and Cochran-Armitage trend test with SAS 9.4.

**Results:**

Approximately 400,000 HIV screening tests were performed at PHCs annually, which remained unchanged in the past 10 years. The proportion of anonymous testing increased from < 3.0% before 2014 to 4.8% in 2014 and 6.1% in 2015. While the number of HIV cases increased, the number of anonymous HIV-positive test results per 10,000 tests decreased from 68.8 in 2010 to 41.8 in 2015. The HIV prevalence among the suspected was approximately 20.0 per 10,000 test takers before 2014, which steeply increased to 71.6 in 2015. Those with suspected HIV were predominantly men, aged 20 years, foreigners, and metropolitan city dwellers in the last 6 years. The high prevalence of persons with suspected HIV resulted in a doubling of HIV prevalence at PHCs between 2014 and 2015.

**Conclusions:**

Anonymous and Suspected, which were driven by similar motives, impacted each other. Increase in HIV prevalence among the suspected led to a higher HIV prevalence among all test takers in PHCs and higher proportions of HIV infection nationwide, which could be attributed to the increase in the number of anonymous tests performed in PHCs. HIV positivity among the anonymous and HIV prevalence among the suspected are key indexes of the national HIV surveillance in Korea.

## Background

While new cases of human immunodeficiency virus (HIV) infection are declining globally, 36.7 million people were diagnosed with HIV, and around 1.8 million new cases are reported annually. Worldwide, 940,000 people died of acquired immune deficiency syndrome (AIDS) in 2017, indicating that the public health burden associated with HIV infection is extremely high [1]. In 2014, the Joint United Nations Program on HIV/AIDS set the 90–90-90 target, which should be achieved by 2020; an interim check on this goal in 2016 showed a value of 70–77-82. The first goal (90%) was related to the proportion of people with HIV who were aware of their HIV status; the first goal is linked to the second and third goals. Seven countries have currently achieved the first goal, including Malaysia and Thailand [2].

In Korea, since the first case of HIV infection was diagnosed in 1985, approximately 14,000 people have been diagnosed with HIV infections between 1985 and 2015, and the number of persons newly diagnosed with HIV infection continues to increase annually. The annual number of HIV cases across Korea has increased to 1000 cases since 2013. Over 99% of HIV infection cases in Korea are caused by sexual contact, with a male to female sex ratio of 9.7 to 1.0; of the total HIV-infected persons diagnosed in Korea, 10% are from foreign countries [3]. HIV infection is predominantly detected through HIV tests performed in hospitals, public health centers (PHCs), and blood banks [4]. In Korea, blood donors at blood banks and donors in hospitals undergo mandatory HIV testing prior to transfer of bodily fluids or body parts [5]. At risk groups for sexually transmitted infection (STI), including commercial sex workers, prisoners, and military enlistees undergoing medical exams, are routinely screened for HIV. Individuals comprising the general population voluntarily undergo HIV testing during routine health checkups. Approximately 10 million HIV screening tests are performed annually in Korea [6, 7]. A total of 253 PHCs in Korea conduct approximately 400,000 HIV screening tests annually, accounting for 3–4% of the total proportion of HIV screening tests in Korea [8].

In a previous study on trends in HIV prevalence among overall visitors to PHCs from 2000 to 2009, we reported that the proportion of new cases of HIV infection detected at PHCs each year had increased to 18% of total new HIV infection cases [9]. Although the proportion of HIV screening tests performed at PHCs has recently decreased, the proportion of HIV cases detected at PHCs has increased to 20%. To identify the reasons for this increasing proportion of new HIV cases detected at PHCs, it is important to monitor the magnitude of the HIV testing and to examine the patterns of newly diagnosed cases of HIV infection at PHCs. The objective of this study was to determine the magnitude and changes in numbers of anonymous HIV test takers (the anonymous), which refers to individuals who voluntarily undergo HIV tests but do not reveal their identity, and analyze changes in anonymous HIV test patterns at PHCs. We then compared the trend of the anonymous with the trend of a similar group: those with suspected HIV infection who voluntarily underwent HIV testing and revealed their identity (the suspected).

## Methods

### HIV test data collection from the national HIV surveillance system at PHCs

Since 1985, various HIV testing policies have been formulated for early HIV detection in Korea. Initially, government-led HIV testing policies mainly focused on mandatory screening for high risk HIV-infected groups. This was subsequently expanded to include mandatory testing in groups of workers sanitation related employment, such as food industry sanitation workers. In October 1989, an anonymous testing scheme was initiated at PHCs in Korea. However, since changes were made to the voluntary testing scheme as part of the HIV testing policy in 1998, changes have been observed in overall HIV testing patterns [10–12]. In March 2008, the Prevention Act of Acquired Immunological Deficiency Syndrome amended the legislation pertaining to an anonymous surveillance system for HIV/AIDS prevention, which was first enacted in 1987 [6]. Currently, the Korean government is formulating HIV-prevention policies to promote counseling and anonymous screening tests for early HIV detection.

PHCs are one of the main screening test sites for HIV in Korea, and refer HIV-positive samples, which were evaluated using enzyme-linked immunosorbent assay (ELISA), particle agglutination (PA), and rapid tests, to the local Institute of Health & Environment (IHE) centers for confirmation of HIV infection. These confirmatory tests were performed using antigen ELISA, antibody ELISA, PA, and Western blots. Confirmed results were reported to the division of HIV and tuberculosis (TB) control of the Korea Centers for Disease Control and Prevention (KCDC) and were recorded in the HIV database of the KCDC [13]. HIV test results were conveyed to the test taker directly at the PHC, by telephone, or through a web interface. Of note, the anonymous HIV test results were communicated to the test taker using a personal telephone access code.

PHCs manage the data related to HIV testing through an electronic medical records system, the Health Care Information System (HCIS). The HCIS program was initiated in 2000 and was expanded to all PHCs in 2005 [14]. We collected the HIV testing data from the HCIS of 253 PHCs between 2000 and 2015. The following data were collected: institutional code, specimen code, sex, year of birth, reason for HIV testing, HIV screening test results, differentiation code, and confirmatory HIV test code. If the result of a HIV screening test was positive, a confirmatory test code was assigned to the IHE center referral for confirmatory HIV testing. The confirmatory test code comprises the referral year, referral institution, and referral order. The differentiation code was a parameter used for the identification of a person’s frequency of test taking within a year. In these codes, the use of personal information was avoided to preserve patient privacy. These data variables were accounted for in the previous manuscripts [9, 14]. Data on HIV-positive cases were collected from the Division of HIV and TB control of the KCDC. These data included sex, year of birth, the reason for HIV testing, and confirmatory HIV test code. Data from HIV testing and data on the HIV-positive cases were matched using confirmatory HIV test codes. In the data recorded manually before the installation of the HCIS program, only the number of HIV tests without variables was obtained.

### Participants

The reasons for HIV testing were classified into 13 groups according to occupation, health status, or motive for testing: health checkup, medical certificate, prenatal checkup, referral by a doctor, the suspected, tuberculosis patient, prisoner, partner of a person with HIV infection, commercial sex worker, bar employee, tea-room employee, massage parlor employee, and the anonymous [14]. The anonymous refers to individuals who voluntarily underwent HIV testing to evaluate their HIV status after risk events but did not reveal their identity. By contrast, the suspected refers to individuals with suspected HIV infection who voluntarily underwent testing only to detect HIV infection and revealed their identity. Data were collected on the anonymous tests performed from 2000 to 2015 and the 12 reasons for HIV testing. If the anonymous test takers were informed that their HIV test results were positive, their reason for HIV testing could be changed from “anonymous” to “suspected” to facilitate referral for public services at PHCs.

### Statistical methods

The annual HIV prevalence among all HIV test takers except the anonymous was defined as the number of confirmed HIV cases per 10,000 HIV test takers, which were calculated using a differentiation code. The frequency of HIV testing for each test taker, that is, the number of repeat HIV testing per person, was measured every year. In case the test taker provided two or more reasons for undergoing HIV testing within 1 year, the person was classified into the reason for testing of the test that provided positive HIV results. If a test taker who cited two or more reasons had negative test results for all tests, the reason for testing was classified as follows: HIV infection suspected group (the suspected or referral by doctor), HIV test recommended group (patients with tuberculosis, prisoner), STI risk group (commercial sex worker, bar employee, tea-room employee, or massage parlor employee), or general group (health checkup, medical certificate, or prenatal checkup). To analyze the HIV prevalence, data on the anonymous were excluded, because the frequency of anonymous tests per person within 1 year could not be measured. Therefore, the annual HIV positivity of the anonymous was calculated and defined as the number of HIV-positive cases per 10,000 anonymous HIV tests [14].

The trends in HIV prevalence of HIV test takers and HIV positivity of the anonymous at PHCs were assessed through a series of cross-sectional annual analyses [9]. We analyzed HIV prevalence among the test takers by sex (male, female), age (< 20, 20–29, 30–39, 40–49, 50–59, ≤60 years), nationality (Korean, foreigner), and region (metropolitan cities, small cities, or rural areas). HIV positivity of the anonymous was analyzed only by region. Data with missing values (about 0.7%) were excluded from the analysis of HIV prevalence. To assess the difference in HIV prevalence by epidemiological variables, we conducted a multivariate logistic regression analysis (sex, age, nationality, and region). Sixteen-year trends in the HIV prevalence and HIV positivity of the anonymous were analyzed using a Cochran-Armitage trend test [15]. All statistical analyses were performed using SAS 9.4 and R. Ethical approval was obtained from the KCDC Institutional Review Board (approval no. 2016-07-06-PE-A).

## Results

### Anonymous HIV testing status at PHCs

Approximately 400,000 HIV tests were conducted annually across 253 PHCs nationwide, and the HIV test scale remained unchanged during the last 10 years. Of the HIV tests performed at the PHCs, the annual proportion of HIV tests of the anonymous and the suspected was less than 10% until 2008; this value increased to 11–16% from 2009 (*P* < 0.001). The annual proportion of anonymous tests accounted for 1–3% of the total HIV tests performed at the PHCs; this value increased by 2–3 times in 2014 and 2015, to values of 4.8 and 6.1%, respectively. The annual proportion of HIV tests of the suspected increased from 5 to 13% from 2000 to 2013 and decreased to 7.8 and 8.1% in 2014 and 2015, respectively (Table 1).

**Table 1:** Changes in the status of HIV testing at public health centers in Korea from 2000 to 2015

Year	Total	Subtotal		Anonymous		Suspected	
	Total HIV tests* No of tests by^ HCIS	N	%^§^	n	%^§^	n	%^§^
2000	428,698 129,607	9410	7.2	2497	1.9	6913	5.3
2001	487,134 229,006	15,440	6.7	4157	1.8	11,283	4.9
2002	484,028 296,414	20,712	7.0	5864	2.0	14,848	5.0
2003	466,747 349,488	29,143	8.4	6592	1.9	22,551	6.5
2004	496,890 435,101	32,920	7.6	6822	1.6	26,098	6.0
2005	353,440 353,440	31,728	9.0	9877	2.8	21,851	6.2
2006	411,017 411,017	34,610	8.5	10,946	2.7	23,664	5.8
2007	396,943 396,943	37,903	9.5	9609	2.4	28,294	7.1
2008	391,486 391,486	38,706	9.9	8595	2.2	30,111	7.7
2009	395,771 395,771	45,217	11.5	8977	2.3	36,240	9.2
2010	378,590 378,590	52,130	13.8	9775	2.6	42,355	11.2
2011	401,320 401,320	64,771	16.2	11,913	3.0	52,858	13.2
2012	423,496 423,496	64,118	15.2	12,157	2.9	51,961	12.3
2013	439,173 439,173	66,453	15.1	11,972	2.7	54,481	12.4
2014	416,661 416,661	52,356	12.6	19,920	4.8	32,436	7.8
2015	429,795 429,795	61,329	14.2	26,322	6.1	35,007	8.1

Anonymous refers to individuals who voluntarily underwent HIV testing to determine their HIV status, but did not reveal their identity

Suspected refers to individuals who voluntarily underwent HIV testing only to detect HIV infection and revealed their identity

*The total HIV tests conducted at PHCs were collected by the HCIS, including documentation from PHCs

^The number of tests based on HCIS data from 72 PHCs in 2000, 114 PHCs in 2001, 150 PHCs in 2002, 186 PHCs in 2003, and 210 PHCs in 2004

The HCIS system has been implemented in all PHCs nationwide since 2005

^§^The proportion of HIV tests among the number of tests performed by the HCIS per year

*HCIS* Health Care Information System, *HIV* human immunodeficiency virus

### Changes in HIV positivity among anonymous test takers from 2000 to 2015

**Fig. 1:**
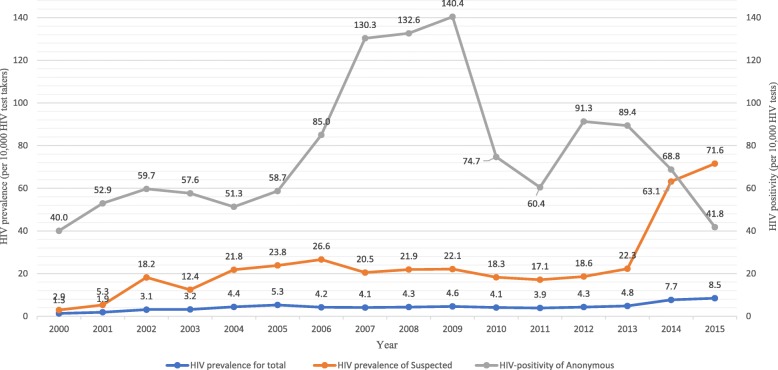
Trends in HIV prevalence by HIV test takers and HIV positivity among anonymous test takers at public health centers in Korea, 2000–2015. Total refers total HIV test takers at public health centers in 1 year. The anonymous refers to individuals who voluntarily underwent HIV testing to determine their HIV status, but did not reveal their identity. The suspected refers to individuals who voluntarily underwent HIV testing to detect HIV infection and revealed their identity. HIV prevalence refers to the number of HIV-infected persons per 10,000 HIV test takers. HIV positivity refers to the number of HIV-positive cases per 10,000 HIV anonymous HIV tests

The HIV positivity among the anonymous was higher than the prevalence among the suspected and total test takers from 2000 to 2014. The HIV positivity among the anonymous and the HIV prevalence among the persons with suspected HIV infection (the suspected) was inverted in 2015. The HIV positivity increased from 40.0 per 10,000 tests in 2000 to 140.4 per 10,000 tests in 2009 and then decreased in 2010. From 2013, the HIV positivity among the anonymous decreased sharply: 89.4 per 10,000 tests in 2013, 68.8 per 10,000 tests in 2014, and 41.8 per 10,000 tests in 2015 (*P* < 0.001). The prevalence among the suspected was about four- to five-fold higher than that of total test takers at PHCs. The HIV prevalence among the suspected in 2000 and 2001 was lower than 10.0 per 10,000 persons, but increased to over 20.0 per 10,000 persons from 2004 to 2013, with no significant change in HIV prevalence in the same period (10 years); in 2014 and 2015, these values more than tripled to 63.1 and 71.6 per 10,000 persons, respectively. The annual HIV prevalence among total test takers at PHCs did not significantly change from 3.0 to 5.0 per 10,000 persons, except in 2000 and 2001; however, it increased sharply to 7.7 and 8.5 per 10,000 persons in 2014 and 2015, respectively. (Fig. 1).

### Characteristics of HIV prevalence among persons with suspected HIV infection

To identify the characteristics of HIV prevalence among the suspected in 2014 and 2015, the relevant data were analyzed by sex, age, nationality, and region from 2010 to 2015. While the sex ratio of the suspected was generally balanced, the HIV prevalence within 6 years was higher in men than in women. HIV prevalence in men increased sharply in 2014 and 2015 (*P* < 0.001). From 2010 to 2015, the age distribution of the suspected was highest among those in their 20s and 30s, and the prevalence of this age group increased steeply across the six study years (P < 0.001). In 2014 and 2015, persons aged 20 years, 40 years, and over 80 years had higher HIV prevalence than other age groups. Among the suspected, 5000–6000 foreigners were tested in 2010 and 2011, but the prevalence of tested foreigners decreased in 2012; however, the HIV prevalence started increasing sharply in this population in 2013. Locally, the prevalence among the suspected from small cities or rural areas who underwent HIV testing was higher than those from metropolitan cities. By contrast, HIV prevalence was higher in the metropolitan cities than in small cities or rural areas, with a sharp increase in 2014 and 2015 (Table 2).

**Table 2:** Characteristics of HIV prevalence (per 10,000) among persons with suspected HIV infection who voluntarily underwent HIV testing and revealed their identity at public health centers in Korea from 2010 to 2015

Category	2010			2011			2012			2013			2014			2015		
	N	Prevalence	aOR	N	Prevalence	aOR	N	Prevalence	aOR	N	Prevalence	aOR	N	Prevalence	aOR	N	Prevalence	aOR
	(HIV+)	(95% CI)	(95% CI)	(HIV+)	(95% CI)	(95% CI)	(HIV+)	(95% CI)	(95% CI)	(HIV+)	(95% CI)	(95% CI)	(HIV+)	(95% CI)	(95% CI)	(HIV+)	(95% CI)	(95% CI)
Total	39,430	18.3		49,660	17.12		49,428	18.61		52,079	22.27		30,727	62.16		33,402	71.55	
	(72)	(14.0–22.5)		(85)	(13.5–20.8)		(92)	(14.8–22.4)		(116)	(18.2–26.3)		(191)	(53.4–70.9)		(239)	(62.5–80.6)	
Sex																		
Female	20,744	2.1	1	22,932	2.1	1	23,491	1.3	1	25,541	6.3	1	16,054	4.4	1	15,319	7.8	1
	(4)	(25.0–40.6)		(5)	(0.0–4.2)		(3)	(0.0–2.7)		(16)	(3.2–9.3)		(7)	(1.1–7.6)		(12)	(3.4–12.3)	
Male	18,686	32.8	19.0	26,728	32.8	13.8	25,937	34.3	27.1	26,538	37.7	6.1	14,673	125.4	29.5	18,083	69.9	16.4
	(68)	(0.0–4.2)	(6.9–52.1)	(80)	(25.0–40.6)	(5.6–34.0)	(89)	(27.2–41.4)	(8.6–85.7)	(100)	(30.3–45.1)	(3.6–10.3)	(184)	(107.4–143.4)	(13.9–62.7)	(227)	(60.9–79.0)	(9.2–29.4)
Age Group (y)																		
< 20	2441	0	0	4655	2.2	0.3	865	23.1	7.4	3322	3	1.4	1563	19.2	1.6	1937	15.5	0.9
	(0)	(0.0–0.0)	(0.0–0.0)	(1)	(0.0–6.4)	(0.0–2.7)	(2)	(0.0–55.1)	(1.4–40.7)	(1)	(0.0–8.9)	(0.1–15.6)	(3)	(0.0–40.9)	(0.4–7.1)	(3)	(0.0–33.0)	(0.2–3.7)
20–29	13,171	17.5	1.5	14,676	20.4	2.5	11,126	34.2	11.0	15,185	31.6	14.9	9839	91.5	7.7	11,395	97.4	5.7
	(23)	(10.3–24.6)	(0.5–4.3)	(30)	(13.1–27.7)	(1.0–6.5)	(38)	(23.3–45.0)	(3.9–30.9)	(48)	(22.7–40.5)	(3.6–61.3)	(90)	(72.6–110.)	(2.8–21.1)	(111)	(79.4–115.4)	(2.3–14.1)
30–39	10,276	22.4	1.9	10,820	19.4	2.4	10,680	25.3	8.1	11,028	26.3	12.4	8848	46.3	3.9	9316	68.7	4.0
	(23)	(13.2–31.5)	(0.7–5.5)	(21)	(11.1–27.7)	(0.9–6.3)	(27)	(15.8–34.8)	(2.8–23.3)	(29)	(16.7–35.9)	(3.0–51.9)	(41)	(32.2–60.5)	(1.4–10.8)	(64)	(51.9–85.5)	(1.6–10.0)
40–49	6083	23	2.0	7640	27.5	3.4	6874	17.5	5.6	6734	34.2	16.1	3719	83.4	7.0	4312	88.1	5.2
	(14)	(11.0–35.1)	(0.6–6.0)	(21)	(15.7–39.2)	(1.3–9.0)	(12)	(7.6–27.3)	(1.8–17.4)	(23)	(20.2–48.1)	(3.8–68.4)	(31)	(54.1–112.6)	(2.5–20.0)	(38)	(60.2–116.0)	(2.0–13.2)
50–59	4051	19.8	1.7	5732	12.2	1.5	7122	12.6	4.0	6458	20.1	9.4	3457	63.6	5.3	3573	50.4	2.9
	(8)	(6.1–33.4)	(0.5–5.6)	(7)	(3.2–21.3)	(0.5–4.7)	(9)	(4.4–20.9)	(1.3–13.1)	(13)	(9.2–31.1)	(2.1–41.9)	(22)	(37.1–90.1)	(1.8–15.5)	(18)	(27.2–73.6)	(1.1–7.9)
60≤	3409	11.7	1	6137	8.2	1	12,762	3.1	1	9353	2.1	1	3301	12.1	1	2870	17.4	2
	(4)	(0.2–23.2)		(5)	(1.0–15.3)		(4)	(0.1–6.2)		(2)	(0.0–5.1)		(4)	(0.2–24.0)		(5)	(2.2–32.7)	
Nationality																		
Korean	33,878	20.1	1	43,322	18.7	1	48,049	18.5	1	50,576	21.4	1	29,690	61.3	1	32,325	68.1	1
	(68)	(15.3–24.8)		(81)	(14.6–22.8)		(89)	(14.7–22.4)		(108)	(17.3–25.4)		(182)	(52.4–70.2)		(220)	(59.1–77.0)	
Foreign	5552	7.2	0.4	6338	6.3	0.3	1379	21.8	1.2	1503	53.2	2.5	1037	86.8	1.4	1077	176.5	2.7
	(4)	(0.1–14.3)	(0.1–0.9)	(4)	(0.1–12.5)	(0.1–0.9)	(3)	(0.0–46.3)	(0.4–3.7)	(8)	(16.4–90.0)	(1.2–5.2)	(9)	(30.3–143.2)	(0.7–2.8)	(19)	(97.8–255.1)	(1.7–4.4)
Region																		
Metropolis	13,188	38.7	4.9	15,736	34.3	3.8	11,419	52.5	6.3	15,181	49.4	4.5	8882	139.6	4.7	10,701	162.6	5.8
	(51)	(28.1–49.3)	(2.9–8.1)	(54)	(25.2–43.5)	(2.4–5.9)	(60)	(39.3–65.8)	(4.1–9.7)	(75)	(38.4–60.6)	(3.1–6.6)	(124)	(115.2–164.0)	(3.5–6.3)	(174)	(138.6–186.6)	(4.4–7.8)
Smaller city or rural area	26,242	8	1	33,924	9.1	1	38,009	8.4	1	36,898	11.1	1	21,845	30.7	1	22,701	28.6	1
	(21)	(4.6–11.4)		(31)	(5.9–12.4)		(32)	(5.5–11.3)		(41)	(7.7–14.5)		(67)	(23.3–38.0)		(65)	(21.7–35.6)	

*HIV* human immunodeficiency virus, *N* Number of HIV test takers, *HIV+* Number of HIV-infected persons, Prevalence: the number of HIV-infected persons per 10,000 HIV test takers, *CI* confidence interval, *aOR* Adjusted odds ratio

## Discussion

Our results show that while the number of anonymous HIV tests increased in Korea, the HIV positivity among the anonymous decreased in 2014 and 2015. Additionally, while the number of the suspected who underwent HIV testing decreased, the HIV prevalence among the suspected increased in the last 2 years. In recent years, the total number of HIV tests performed in the anonymous and the suspected remained unchanged. However, the decision to undergo anonymous testing, which was driven by similar motives in both the anonymous and the suspected, impacted each other. The increase in HIV prevalence among the suspected led to the increase in the HIV prevalence among all test takers at PHCs, which are the primary testing sites of the national HIV surveillance system in Korea.

The number of individuals with newly diagnosed HIV infection has been continuously increasing in Korea, with more than 1000 HIV-infected people reported annually since 2013: 953 people in 2012, 1114 in 2013, 1191 in 2014, and 1152 in 2015. More than 25% of all newly diagnosed HIV infections in Korea were diagnosed through HIV screening tests at PHCs [3]. According to the results of this study, of the new HIV infection cases diagnosed at PHCs, more than 50% were observed in the suspected in 2008; this proportion increased to 71.3 and 77.9% in 2014 and 2015, respectively. Therefore, most of the HIV infections identified at the PHCs were diagnosed in the suspected. This was because the PHCs actively provided various opportunities for HIV testing, including free HIV testing and rapid diagnostic tests for early HIV detection.

The patterns of HIV testing and HIV detection continued to change over the time period of this study. The decrease in HIV positivity among the anonymous after 2009 was possibly due to the active HIV anonymous testing program. Anonymous tests have been conducted in hospitals in Korea since 2008, and the number of anonymous tests at PHCs increased after 2009. In addition, as a result of the expanded program for rapid diagnostic testing used to encourage people to undergo anonymous tests at PHCs, the number of anonymous HIV tests doubled in 2014 and 2015. In a pilot project on rapid diagnostic testing introduced at four PHCs in Seoul in 2014, the number of anonymous tests increased nine-fold and the number of confirmed HIV infection cases increased four-fold [16]. Since then, the number of PHCs that provided rapid diagnostic tests increased to 34; by 2015, the proportion of tests that were anonymous at PHCs in Seoul increased to 80% (a total of 22,114 tests). The proportions of HIV testing and HIV detections of the anonymous and the suspected in Seoul varied dramatically in 2014 and 2015. Due to activation of the anonymous HIV testing program by rapid diagnostic testing at PHCs in 2014, individuals who want to determine their HIV status might need to undergo an anonymous test in order to obtain their HIV test results quickly. Therefore, the number of anonymous tests doubled, and the number of HIV tests performed among the suspected decreased to approximately 40% in 2014 and 2015. Some individuals who were aware of their HIV-positive status by anonymous test revealed their identities to receive welfare benefits, including government-sponsored medical support. Their reason for HIV testing might be reverted on HCIS at PHCs. The number of reverted cases was increased annually and showed a steep increase in the last 2 years: 23 cases in 2010, 16 cases in 2011, 33 cases in 2012, 35 cases in 2013, 42 cases in 2014, and 49 cases in 2015. In 2014 and 2015, HIV positivity among the anonymous decreased and the number of HIV detection among the suspected increased. Therefore, the results of this study demonstrate how changes in the national HIV testing strategy affected the status of HIV detection.

In Europe in 2016, 15% of the 810,000 individuals infected with HIV were not aware of their HIV-positive status, and about 48% of 30,000 newly infected persons (5.9 per 100,000 persons) were diagnosed at a later stage of HIV infection (CD4 < 350 cells/mm^3^) [17]. Therefore, for early HIV detection, the United Kingdom modified the environment or facilities of the examination clinics and improved accessibility to anonymous testing, as well as increasing education and promotional activities. These changes were attributed to a large increase in the number of anonymous HIV test takers who identified as male and homosexual. Consequently, new HIV infections among homosexuals decreased by 29% in London and by 11% outside London in 2015 [18]. In Korea, among men who had sex with men (MSM) and transgenders, HIV voluntary counseling and testing (VCT) to diagnose HIV infection early and to prevent HIV infection has been performed in Seoul and Pusan [19].

In Korea, foreigners accounted for about 10% of the total HIV-positive cases, and this proportion is increasing every year [3]. Our study showed that the prevalence of HIV among foreigners rapidly increased in 2014 and 2015 and was higher than the prevalence among Korean patients. The increase in number of foreigners diagnosed with HIV corresponds to an increase in multicultural families, foreign workers, and tourists. In Europe, a large number patients newly diagnosed with HIV infection were migrants from Africa and other countries [20].

This study has two limitations worth noting. First, as the HCIS was installed in all PHCs in 2005, there may be biases pertaining to the estimated proportions, HIV positivity among anonymous test takers, and the prevalence before 2005, as these were calculated based on data from the 72–210 PHCs with the HCIS. Second, repeat anonymous tests for anonymous test takers and HIV tests for those with suspected HIV were either performed in the same PHC or in other PHCs. It is possible that some test results of the anonymous and the suspected could be of duplicate cases that were included in our analysis.

## Conclusion

This study demonstrated that changes in the national HIV testing strategy affected the status of HIV detection in Korea. In particular, the introduction of the rapid diagnostic tests at PHCs since 2014 attributed to the increase in the number of anonymous tests. People fear the stigma associated with HIV/AIDS, and they prefer to be tested for HIV infection anonymously, which provides quick results without revealing their identity.

As anonymous HIV testing has been expanded to medical institutes since 2008 and HIV self-testing has been performed since 2015, it is impossible to determine the status of anonymous HIV test across Korea. Therefore, HIV positivity among the anonymous and HIV prevalence among the suspected at PHCs are important indexes. Hence, these factors should be continuously monitored and improved in other sites. In the future, more than 90% of Koreans with HIV should be made aware of their HIV status through provision of easier access to anonymous testing. This will aid in the prevention of HIV infection spread in Korea and serve as a foundation to support the treatment of people with HIV infection who do not progress to AIDS through the administration of antiviral drugs and healthcare.

## Data Availability

Not applicable.

## Abbreviations

AIDS: Acquired immune deficiency syndrome
CI: Confidence interval
ELISA: Enzyme-linked immunosorbent assay
HCIS: Health Care Information System
HIV: Human immunodeficiency virus
IHE: Institute of Health & Environment
KCDC: Korea Centers for Disease Control and Prevention
PA: Particle agglutination
PHC: Public Health Center
